# Activation of Protein Kinase G (PKG) Reduces Neointimal Hyperplasia, Inhibits Platelet Aggregation, and Facilitates Re-endothelialization

**DOI:** 10.1038/srep36979

**Published:** 2016-11-11

**Authors:** Ju-Young Kim, Han-Mo Yang, Joo-Eun Lee, Baek-Kyung Kim, Sooryeonhwa Jin, Jaewon Lee, Kyung-Woo Park, Hyun-Jai Cho, Yoo-Wook Kwon, Hae-Young Lee, Hyun-Jae Kang, Byung-Hee Oh, Young-Bae Park, Hyo-Soo Kim

**Affiliations:** 1 National Research Laboratory for Cardiovascular Stem Cell, Seoul National University College of Medicine, Seoul, Republic of Korea; 2 Innovative Research Institute for Cell Therapy, Seoul National University Hospital, Seoul, Republic of Korea; 3 Department of Internal Medicine, Seoul National University Hospital, Seoul, Republic of Korea; 4 Molecular Medicine and Biopharmaceutical Sciences, Seoul National University, Seoul, Korea

## Abstract

In spite of its great success in reducing restenosis, drug-eluting stent (DES) has unfavorable aspects such as stent thrombosis and delayed re-endothelialization. We examined the effects of PKG activation by Exisulind on neointimal formation, platelet aggregation, and re-endothelialization. Exisulind significantly reduced VSMCs viability, cell cycle progression, migration, and neointimal hyperplasia after vascular injury in rat carotid arteries. Interestingly, in contrast to the effect on VSMC viability, Exisulind did not reduce the viability of endothelial cells. Increased PKG activity by Exisulind inhibited PDGF-stimulated phenotype change of VSMCs from a contractile to a synthetic form. Conversely, the use of PKG inhibitor or gene transfer of dominant-negative PKG reversed the effects of Exisulind, resulting in the increased viability of VSMCs and neointimal formation. In addition, Exisulind facilitated the differentiation of peripheral blood mononuclear cells to endothelial lineage via PKG pathway, while inhibiting to VSMCs lineage, which was correlated with the enhanced re-endothelialization *in vivo*. Finally, Exisulind reduced platelet aggregation, which was mediated via PKG activation. This study demonstrated that Exisulind inhibits neointimal formation and platelet aggregation while increasing re-endothelialization via PKG pathway. These findings suggest that Exisulind could be a promising candidate drug of DES for the prevention of restenosis without other complications.

Drug-eluting stents (DES) using anti-proliferative agents such as rapamycin or paclitaxel, have become the most promising method to prevent restenosis after percutaneous coronary intervention (PCI)^1^,^2^. Despite the remarkable success, however, DES has shown unexpected adverse responses such as delayed re-endothelialization and stent thrombosis, as compared to bare-metal stents^2^,^3^. Moreover, high risk subgroups such as diabetics and patients with complex lesions, still show high restenosis rate. Therefore, many attempts have been made to seek another ideal drug for prevention of both restenosis and the adverse effects of DES^4^.

After vascular injury by balloon angioplasty or stent implantation, diverse mechanisms are activated, leading to neointimal hyperplasia. Among them, two mechanisms can be targeted to reduce restenosis. One is to inhibit the proliferation and migration of VSMCs, and the other is to facilitate re-endothelialization^5^,^6^,^7^,^8^,^9^. Thus, it is very important to find an agent that fulfills these two goals. In addition, if a drug could also inhibit platelet aggregation, it would be very helpful because it prevents stent thrombosis associated with DES^10^.

In the responses of VSMCs after vascular injury, a phenotype change is very important in the processes involved with neointimal formation^11^,^12^. Vessel injury converts a contractile type of VSMCs to a synthetic one^12^. Several studies have shown that the phenotype change of VSMC from a contractile to a synthetic form is associated with reduced vascular cGMP-dependent Protein Kinase (Protein Kinase G, PKG)^13^,^14^,^15^. This suggests that PKG plays an important role in modulating the VSMC phenotype and neointimal formation, which are associated with restenosis after stent implantation. Interestingly, PKG is present at high levels in platelets and associated with inhibition of platelet aggregation^16^. Thus, we can assume that drugs that modulate PKG activity could be used as a candidate drug for DES without increasing stent thrombosis.

Among those drugs modulating PKG activity, we chose Exisulind as a potential candidate drug for DES. Exisulind (Sulindac sulfone) is known to activate PKG. Here, we examined the effects of Exisulind on neointimal formation after balloon injury and its mechanisms of action in VSMCs, endothelial cells, and platelets.

## Materials and Methods

Collagenase type II, Elastase, and Exisulind were obtained from Sigma-Aldrich co. (St Louis, MO, USA). PDGF-BB was purchased from R&D systems (Minneapolis, MN, USA). Goat anti-PKG I, rabbit anti-VE-cadherin, mouse anti-osteoponin, rabbit anti-CD31, and goat anti-actin antibodies were obtained from Santa Cruz Biotechnology (Santa Cruz, CA, USA). Rabbit anti-VASP (Ser239), rabbit anti-t-Akt, and mouse anti-p-Akt (Ser473) were purchased from Cell Signaling (Berkely, MA, USA). Mouse anti-calponin antibody was obtained from Sigma-Aldrich co. (St Louis, MO, USA). Mouse anti-α-SMA antibody was purchased from Abcam (Cambridge, MA, USA). The secondary antibodies to each primary antibody were as follows. Anti-mouse IgG HRP conjugated and anti-rabbit IgG HRP conjugated antibodies were obtained from Promega (Madison, WI, USA). Anti-goat IgG HRP conjugated antibody was obtained from Santa Cruz Biotechnology (Santa Cruz, CA, USA). Anti-mouse IgG Alexa flour 488 and anti-rabbit IgG Alexa flour 594 secondary antibodies were purchased from Invitrogen (Carlsbad, CA, USA). Alzet osmotic pump was purchased from Durect corporation (Cupertino, CA, USA).

### Isolation and culture of rat aortic vascular smooth muscle cells (VSMCs) and endothelial cells (ECs)

All animal studies were performed in accordance with the Experimental Animal Committee of the Clinical Research Institute, Seoul National University Hospital (Seoul, Korea). Rat aortic VSMC were isolated and cultured as previously described with minor modifications^17^,^18^. Briefly, segments of both the abdominal and thoracic aorta were obtained from normal adult Sprague-Dawley (SD) rats by careful dissection under sterile conditions. An aortic segment was placed in a culture dish containing Dulbecco’s modified Eagle’s medium (DMEM), then the outer lipid and adventitia were carefully removed under a dissecting microscope. The remaining portion of the media and the intima were cut into an approximately 1.0–1.5 mm thick fine ring across the vessel in a culture dish and washed with fresh culture medium.

The aorta rings were transferred to a trypsinizing flask with serum-free DMEM containing 0.2% collagenase (type II) and shaken at 37 °C for 30 minutes at 100 rpm/min. After 30 minutes, supernatant was collected and neutralized with 10% (V/V) fetal bovine serum (FBS). Cells were harvested and centrifuged at 1800 rpm for 10 min. Harvested cells were resuspended at ~0.1–0.5 × 10^6^ cells per mL in medium (EGM-2 MV Bullet Kit system, Lonza, Walkersville, MD, USA, consisting of endothelial basal medium, 5% fetal bovine serum (FBS), hEGF, VEGF, hFGF, IGF-1, and ascorbic acid) and seeded onto fibronectin (10 μg/mL) coated culture dishes. This enzyme treatment was repeated twice for culture of rat aortic endothelial cells. Remaining vessel segments were resuspended in serum-free DMEM containing 0.2% collagenase (type II) and 1 mg/mL elastase (type IV) for isolation of VSMC. Vessel fragments were shaken at 37 °C for 30 minutes at 100 rpm/min. A cell suspension was obtained and centrifuged at 1800 rpm for 10 min. The cell pellet was resuspended in DMEM/F12 containing 10% FBS and seeded onto a non-coated culture dish. This enzyme treatment was repeated until the segments were completely dissolved. The seeded cells were cultured in the above medium at 37 °C in a humidified atmosphere of 5% CO_2_–95% air. Once the cells had attached, old medium was aspirated and replaced with fresh medium. For the cell subcultures, cells were rinsed twice with phosphate-buffered saline (PBS) and briefly treated with trypsin. Aortic EC showed a typical cobble stone colony under phase-contrast microscopy, and identification of EC was verified by VE-cadherin (Fig. S1A). Aortic VSMCs showed typical “hill-and-valley” growth as viewed under phase-contrast microscopy. The VSMCs were verified by calponin and α-smooth muscle actin staining (Fig. S1B). Cells were studied between passages 5 and 16, during which time the investigated parameters remained stable.

### Isolation and culture of human peripheral blood mononuclear cells

All experiments dealing with humans or human products were conducted with informed consent and approved by the Institutional Review Board (IRB) of Seoul National University Hospital (IRB No. H-0905-032-281). And, all methods were performed in accordance with the approved guidelines and regulations. Mononuclear cells were isolated from peripheral blood by density gradient centrifugation with Histopaque 1077 (Sigma, St. Louis, MO) according to manufacturer’s instructions. Isolated mononuclear cells were resuspended using an EGM-2 BulletKit system and 4 × 10^6^ mononuclear cells per well were seeded on 2% gelatin-coated (Sigma, St. Louis, MO, USA) six-well plates and incubated in a 5% CO_2_ incubator at 37 °C. A first medium change was performed 5 days after seeding and separated into subgroups to be treated with either EGM-2 MV medium with or without PDGF (50 ng/mL) or Exisulind medium (100 μM) with or without PDGF. Thereafter, media were changed every 3 days.

### PKG I retrovirus transduction to rat VSMC

Wild type retroviral vector, and constitutively active and dominant negative mutant forms for PKG Iα and PKG Iß were kindly provided by Dr. Deguchi A. (Columbia University). The mutant forms were: PKG IαS65D, IαK390R, IßS80D, IßK405R and the NH_2_-terminal truncated mutants PKG Iα and PKG Iß^19^. Retrovirus producer cells (293FT cells) were co-transfected with retroviral PKG constructs, pGag-pol and pVSV-G as described previously^19^. After 18 hours, transfection medium was replaced with fresh DMEM/10% fetal bovine serum medium. The retrovirus-containing medium was harvested after another 24 hours and passed through a 0.45 μm syringe filter. The viral supernatant was transfer to a conical ultracentrifuge tube and centrifuged at 25,000 rpm for 2 hours at 4 °C. After ultracentrifugation, the supernatant was discarded and the pellet resuspended in an appropriate volume of TNE buffer (50 mM Tris-HCl, pH 7.4, 100 mM NaCl, 0.5 mM EDTA, filtered through 0.22 μm). Rat VSMCs were infected with the indicated retrovirus encoding PKG mutant form for 18 hours, and then incubated in fresh DMEM/F12/10% fetal bovine serum medium for 72 h^20^.

### Cell viability

Rat VSMCs (7 × 10^4^ cells) and rat EC (3 × 10^4^ cells) were plated onto 24-well plastic tissue culture plates and grown to 25–30% confluence. The cells were then cultured with serum-free DMEM/F12 or EBM-2/2% fetal bovine serum medium for 24 hours to provide serum deprivation conditions. Serum starvation medium was replaced with DMEM/F12 containing Exisulind at concentrations of 100 μM and 250 μM before stimulation with 6 ng/mL rat PDGF-BB in rat VSMC case. For rat EC, EBM-2/2% FBS medium was replaced with EBM-2/5% FBS containing Exisulind at concentrations of 100 μM and 250 μM. Cells were harvested by trypsinization, and cell numbers were determined in triplicate using a hemocytometer (Coulter Electronics, Hialeah, FL) at 24 hours and 48 hours. Cell viability after treatment with Exisulind was evaluated by tryphan blue exclusion assay.

### FACS analysis for Apoptosis and Cell cycle

Rat VSMC (2 × 10^5^ cells/well) and rat EC (2 × 10^5^ cells/well) were plated onto 6 well plates and grown to 70–80% confluence. These cells were cultured in media containing 100 μM or 250 μM Exisulind. After 18~24 hours, cells were harvested, washed twice with PBS, and fixed in 70% ethanol at 4 °C. Fixed cells were stained with propidium iodide (50 μg/mL)/DNase-free RNase (2 units/mL) at room temperature for 1 h and analyzed by FACScan (Becton Dickson) using a ModFit LT software package.

### Cell surface marker analysis by FACS

FACS was used to identify cell-surface antigens. For Exisulind medium-treated human mononuclear cells, FITC-conjugated anti–α-smooth muscle actin (α-SMA) and PE-conjugated anti-human CD31 antibodies were used. Adherent cells were detached with trypsin-EDTA and washed in PBS. For marker staining, cells were incubated with 1 μg/mL of antibody for 30 minutes at 4 °C. After staining, labeled cells were washed with FACS buffer (PBS supplemented with 2% BSA and containing 0.05% NaN_3_). Cells were fixed with 4% paraformaldehyde and analyzed using a FACSCalibur™ flow cytometer (Becton Dickinson).

### Scratch wound healing assay

Rat VSMCs were seeded into 6-well plates and left to grow to subconfluence. At the moment of the experiment, rat VSMC monolayers were scratched with a Cell lifter (Costar, Cambridge, MA, USA), creating a cell-free area (‘scratch-wound’) approximately 1 cm in width. ‘Scratch-wounded’ monolayers were washed twice with DMEM/F12 to remove cell debris, and treated with 100 μM, or 250 μM of Exisulind for 3 days. A defined area of the wound was photographed using phase-contrast microscopy. To remove the effect of cell proliferation, cells were pretreated with 1 M thymidine (Sigma, St. Louis, MO, USA), which was added to the cells incubation medium. This inhibits cell proliferation without killing them. Migration was observed for 6–48 hours. At each time, the culture dish was placed on the stage of an inverted microscope (Olympus, Tokyo, Japan) equipped with a CCD camera. Migration images were analyzed using Image Pro Plus (Media cybernetics, Bethesda, MD, USA).

### Immunofluorescence staining

Rat VSMCs were seeded into coverglass-bottom dish (confocal dish; SPL, Pcheon, Korea) and treated with 100 μM, or 250 μM of Exisulind with or without PKG inhibitor, 8-Rp-cPT-cGMP for 18~24 hours. For Immunofluorescence staining, cells were washed twice with PBS, and fixed with 100% cold methanol for 10 minutes in −20 °C. After washing away the methanol with 0.05% TBS-T 3 times, blocking process was performed with a 1% BSA solution. Immunofluorescence staining was performed using FITC labeled anti-α-smooth muscle actin (α-SMA) and anti-calponin antibodies. Anti-mouse IgG Alexa flour 594 was used as a secondary antibody for calponin staining. Nuclei were stained with DAPI. Specimens were observed using a confocal microscope (Carl Zeiss) or an inverted fluorescent microscope (Olympus).

### Platelet aggregation assay

Fresh rat blood was anti-coagulated with sodium citrate (0.109 M). After centrifuging at 750 rpm for 10 minutes at 4 °C, platelet-rich plasma (PRP) was collected and platelet-poor plasma (PPP) was collected from centrifuged PRP at 3000 rpm for 10 minutes at 4 °C. Platelet aggregation was measured by detecting changes in light transmission after introducing the coagulation agent 5 μM ADP. Platelet aggregation and secretion were recorded in real time using a Chrono-log lumiaggregometer at 37 °C with stirring (1000 rpm). For human platelet, separated human PRP were treated with 100 μM, 250 μM Exisulind and 60 μM cGMP analogues with or without PKG inhibitor, 8-Rp-cPT-cGMP for 10 min, 20 min and 24 hours. 5 μM ADP was also used with coagulation agent.

### Western blot analysis

Rat VSMCs and rat ECs were treated with 100 μM, or 250 μM of Exisulind with or without PKG inhibitor, 8-Rp-cPT-cGMP for 18 hours. Rat VSMCs and rat ECs were washed in cold PBS and harvested by scraping in lysis buffer. The lysis buffer contained 50 mM Tris-HCl (pH 7.2), 250 mM NaCl, 1% NP-40, 0.05% SDS, 2 mM EDTA, 0.5% deoxycholic acid, 10 mM β-glycerophosphate, 1 mM vanadate and 1 mM phenyl methyl sulfonyl fluoride (PMSF). One tablet of protease inhibitor cocktail complete mini (Roche, Indianapolis, IN) per 10 mL of lysis buffer, vanadate and PMSF were added just before use. After harvested samples were centrifuged at 15,000 rpm in 4 °C for 30 minutes, the supernatant was transferred into a clean tube. Protein concentrations of whole cell extracts were determined with BCA protein assay kit (Pierce, Rockford, IL, USA). The extracts (25 μg protein) were separated by SDS–PAGE gel, and the procedures described previously were performed^21^ with slight modifications.

### Rat carotid injury model and BrdU incorporation assay

All animal experiments were performed after receiving approval from the Institutional Animal Care and Use Committee (IACUC) of Clinical Research Institute in Seoul National University Hospital and complied with the National Research Council (NRC) ‘Guidelines for the Care and Use of Laboratory Animals. Female Sprague-Dawley rats (12 weeks old, weighing 280–310 g) from Daehan Biolink (Chungbuk, South Korea) were maintained on Rat & Mouse Cubes and fed with standard pellet feed and water ad libitum. Under Xylazine (5 mg/kg IP, Yuhan Corp, Bayer Korea) and ketamine hydrochloride (50 mg/kg IP; Yuhan Corp. Bayer Korea) induced anesthesia, the right external carotid arteries were exposed and ligated. The common carotid arteries were denuded of endothelium by the intraluminal passage of a 2 F arterial catheter (Baxter Healthcare Corp). This was passed to the proximal common carotid artery and withdrawn in the inflated state 5 times and connected to a primed osmotic minipump (Alzet Corporation, Palo Alto, California, USA). For drug administration, Exisulind (0.5 mg/kg/day) were infused into the animals using an osmotic pump for 2 weeks after balloon injury (n = 10). Control rats (n = 10) were administered a DMSO solution in the same manner. The random method for the allocation of rats was used. For analysis of vascular proliferation, intraperitoneal infusion of bromodeoxyuridine (BrdU) (100 mg/kg) was given 24 hours before euthanization on day 7 after balloon injury.

### Tissue preparation, morphometric analysis and immunohistochemistry

Rats were euthanized 3, 7 and 14 days after the injury. The carotid arteries were fixed by 4% paraformaldehyde for overnight. The tissues were then embedded in paraffin. For morphometric analysis, 4 μm thick-sections were stained with elastica van Gieson. The area of residual lumen, the neointimal and the media, were assessed using a digital image-analysis system (Image-Pro Plus 4.5 software). All data were quantified on more than 5 sections/rat with an average of 5 high power fields/section. Moreover, All analyses were performed by at least two blinded and independent observers. For immunohistochemical staining and immunofluorescent staining, anti-PKGI, anti- proliferating cell nuclear antigen (PCNA), anti-α-SMA, anti-Calponin, anti-OPN and anti-CD31 antibodies were applied. Polyclonal secondary antibodies conjugated with avidin-biotin–horseradish peroxidase (Santa Cruz) and were used for signal detection. To assess the degree of apoptosis, terminal deoxynucleotidyl transferase–mediated dUTP-biotin nick end-labeling (TUNEL) staining was applied using an Apoptag kit (Intergen, Purchase, NY, USA).

### Statistical analysis

Data were analyzed using SPSS version 12.0. Results were expressed as the mean ± SEM unless specified otherwise. Continuous variables were tested for normal distribution with the Shapiro-Wilk test. Differences between two groups were determined using the paired or unpaired t-test if normal distribution could be assumed. Differences involving multiple groups were first tested by one-way ANOVA, followed by post-hoc analysis with the Bonferroni method adjusting for multiple comparison. *P* < 0.05 was considered statistically significant.

## Results

### Exisulind inhibited neointimal hyperplasia after angioplasty *in vivo*

**Figure 1:**
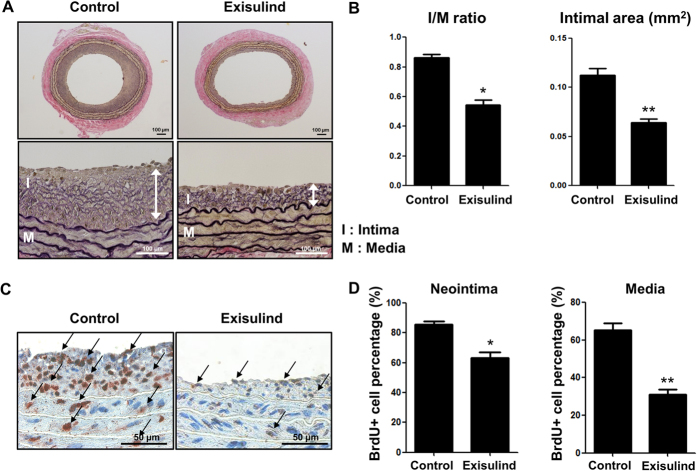
Effects of Exisulind on neointimal formation after balloon injury in rat carotid arteries. (**A**) Representative figures of the vessels at 2 weeks after injury. Exisulind treatment significantly reduced neointimal hyperplasia compared to vehicle-treated group. I = Intima, M = Media. Scale bar = 100 μm. (**B**) Quantification graphs of Intima to Media (I/M) ratio and intimal area at 2 weeks after injury. This graph shows markedly reduced I/M ratio and intimal area in Exisulind-treated group (n = 10 per group). *P < 0.01 vs. control, **P < 0.01 vs. control. (**C,D**) Representative figures and quantification of vascular proliferation performed at day 7 after arterial injury by BrdU incorporation in rats. Exisulind treatment reduced BrdU-positive cells compared to vehicle-treated group. *P < 0.01 vs. control, **P < 0.01 vs. control.

First, we examined whether Exisulind could suppress neointimal formation after vascular injury *in vivo*. In the rat carotid balloon injury model, administration of Exisulind significantly reduced the development of neointimal hyperplasia at 2 weeks, compared with the control group (Fig. 1A). Quantitative analysis showed a significant reduction in both Intima/Media ratio (vehicle-treated group vs. Exisulind-treated group, 0.87 ± 0.02 vs. 0.50 ± 0.02, *P* < 0.01, n = 10 per group) and Intimal area (vehicle-treated group vs. Exisulind-treated group, 0.11 ± 0.008 vs. 0.06 ± 0.003, *P* < 0.01, n = 10 per group) (Fig. 1B). In terms of vascular proliferation, Exisulind treatment significantly reduced the incorporation rate of BrdU compared to control group in both intima and media (vehicle-treated group vs. Exisulind-treated group, 87.01 ± 1.46% vs. 61.84 ± 4.09%, *P* < 0.01 for intima; 65.48 ± 3.78% vs. 28.91 ± 1.85%, *P* < 0.01 for media) (Fig. 1C,D).

### Exisulind reduced VSMC viability but not Endothelial Cell (EC) viability

**Figure 2:**
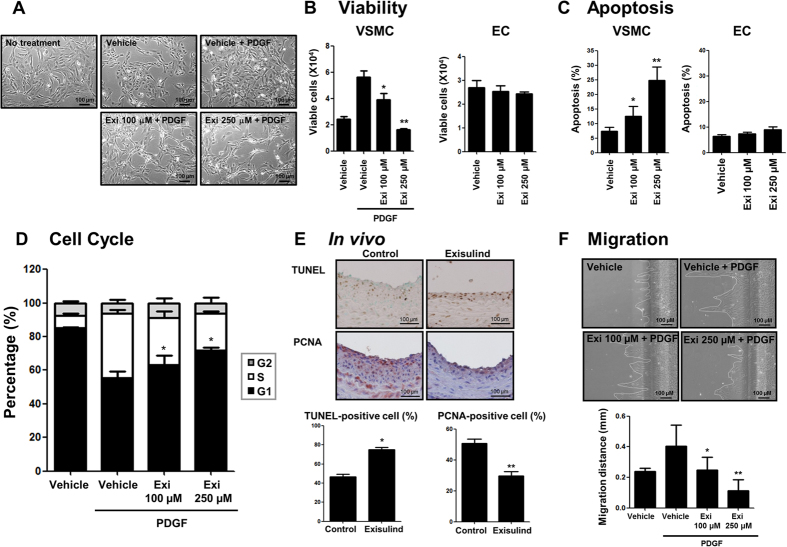
Effects of Exisulind on proliferation or apoptosis of VSMCs and ECs *in vitro* and *in vivo*. (**A**) Morphologic changes of VSMCs when treated with each concentration of Exisulind for 24 hours. Scale bar = 100 μm. Exi = Exisulind. (**B**) Tryphan blue assay showed that Exisulind reduced the viability of VSMCs in a dose-dependent manner. *P = 0.04 vs. PDGF-treated group, **P < 0.01 vs. PDGF-treated group. However, Exisulind did not reduce the viability of ECs, even at relatively high concentration (250 μM). (**C**) Flow cytometry analysis shows that Exisulind increased apoptotic cell fractions in a dose-dependent manner in VSMCs. *P = 0.19 vs. vehicle group, **P < 0.01 vs. vehicle group. However, the apoptotic fraction for EC was not affected by Exisulind treatment. The data are presented as mean + SEM of four to ten independent experiments. (**D**) Cell cycle analysis shows that Exisulind blocked PDGF-induced cell-cycle progression of VSMCs to S phase in a dose-dependent manner. Black bar = G0/G1 phase, gray bar = G2 phase, white bar = S phase. *P < 0.01 vs. vehicle in terms of G1 phase. (**E**) Immunohistochemical staining for TUNEL or PCNA expressions in the carotid artery wall in the vehicle-treated group (left panel) and Exisulind-treated group (right panel). Exisulind treatment increased TUNEL-positive cells and decreased PCNA-positive cells in the vessels. Scale bar = 100 μm. Quantification graph of TUNEL- or PCNA- positive cells suggests that Exisulind increased apoptosis and decreased proliferation in VSMCs. *P < 0.01 vs. control group, **P < 0.01 vs. control group, Scale bar = 100 μm. (**F**) Scratch wound migration assay shows that Exisulind treatment significantly reduced VSMC migration. Scale bar = 100 μm. *P = 0.04 vs. PDGF group, **P = 0.02 vs. PDGF group.

Because Exisulind is known to inhibit cell proliferation or induce apoptosis for some types of carcinoma cells, we evaluated the effect of Exisulind on rat VSMC and EC viability^22^,^23^,^24^. As shown in Fig. 2A, Exisulind-treated VSMCs showed morphological changes from a synthetic to a contractile form, finally showing the decrease of cell number with a higher concentration of Exisulind. Exisulind treatment reduced the viability of VSMCs in tryphan blue exclusion assay, but did not change EC viability (Fig. 2B). To evaluate the contributions of apoptosis and cell cycle arrest to the reduction in VSMC number, the effect of Exisulind on cell cycle and apoptosis was tested. Exisulind significantly increased apoptosis of VSMCs (Fig. 2C) and arrested the cell cycle of VSMC at the G1 phase (Fig. 2D). In contrast, Exisulind did not increase the fraction of apoptosis in ECs (Fig. 2C). Moreover, *in vivo* study showed that Exisulind treatment reduced VSMC proliferation and increased VSMC apoptosis, indicating the same results in the previous *in vitro* studies (Fig. 2E). In addition, scratch wound migration assay demonstrated that Exisulind significantly inhibited the migration capacity of VSMC (Fig. 2F).

### Exisulind regulated PKG activity and modulated VSMC phenotype

**Figure 3:**
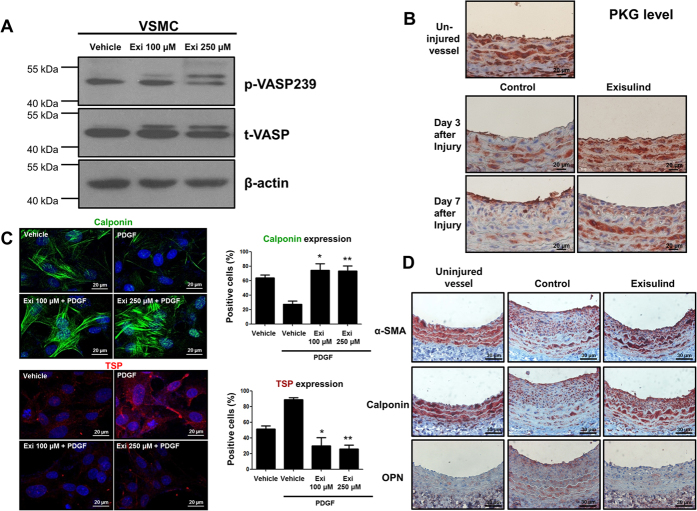
Effects of Exisulind on PKG activity and VSMC phenotype. (**A**) Western blot analysis shows that Exisulind treatment increased VASP phosphorylation (a substrate of PKG) in a dose-dependent manner. p-VASP 239 = phospho-VASP at serine239, t-VASP = total VASP, Exi = Exisulind. (**B**) Immunohistochemical staining for PKG in rat carotid arteries. After balloon injury, PKG level was decreased. Compared with vehicle-treated group, Exisulind treatment reversed the decreased level of PKG induced by vascular injury. Scale bar = 20 μm. (**C**) Immunofluorescence staining for calponin and thrombospondin. PDGF treatment reduced the level of calponin (a marker for the differentiated contractile form of VSMC) and elevated the level of thrombospondin (a marker of synthetic from). Exisulind treatment reversed the effect of PDGF. Scale bar = 20 μm. Exi = Exisulind. *P < 0.01 vs. PDGF group, **P < 0.01 vs. PDGF group in terms of calponin level; *P < 0.01 vs. PDGF group, **P < 0.01 vs. PDGF group in terms of thrombospondin level. The data are presented as mean + SEM of four to five independent experiments. (**D**) Immunohistochemical staining for calponin, α-SMA, and osteopontin. The uninjured vessels showed the high level of α-SMA and calponin, but low level of osteopontin. The arterial wall at 2 weeks after injury showed that Exisulind treatment increased the expression of α-SMA and calponin (a contractile form marker) and decreased the level of osteopontin (a synthetic form marker). Scale bar = 20 μm. OPN = osteopontin.

Western blot analysis of phospho-Vasodilator-stimulated phosphoprotein (VASP) (a substrate of PKG) showed that Exisulind treatment increased the activity of PKG (Fig. 3A). Moreover, *in vivo* study showed that Exisulind treatment upregulated PKG level which had been decreased by vascular injury (Fig. 3B). It is known that PKG activity is associated with the phenotypic changes of VSMC^13^,^14^,^15^. Based on these facts, we hypothesized that Exisulind could modulate the VSMC phenotype. The level of calponin, one of the markers for the differentiated contractile form of VSMC, was examined by immunofluorescence staining. PDGF-BB stimulation reduced calponin expression, which was dramatically reversed by Exisulind treatment (Fig. 3C). In case of thrombospondin, a marker of synthetic form, we could see the opposite change. This suggests that Exisulind can modulate the VSMC phenotype. Immunohistochemistry for these markers in both the uninjured and injured vessel wall showed the consistent results (Fig. 3D).

### The effect of Exisulind on VSMCs and neointimal formation was mediated through PKG pathway

**Figure 4:**
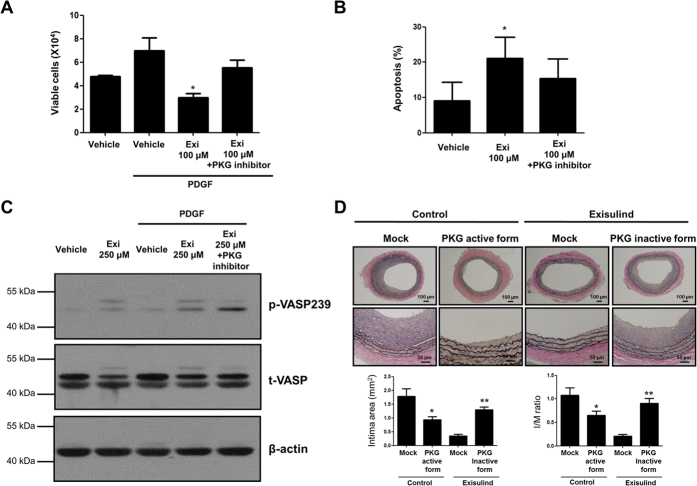
Effects of Exisulind mediated through PKG pathway *in vitro* and *in vivo*. (**A,B**) The graphs show that PKG inhibitor (8-Rp-cPT-cGMP: 20 μM) reversed the effects of Exisulind on VSMCs viability and apoptosis. Exi = Exisulind. *P = 0.02 vs. PDGF group in (**A**), *P = 0.04 vs. vehicle group in (**B**). The data are presented as mean + SEM of five independent experiments. (**C**) Western blot assay demonstrates that the increased activity of PKG was reversed by PKG inhibitor (8-Rp-cPT-cGMP: 20 μM) treatment. p-VASP239 = phospho-VASP at serine239, t-VASP = total VASP, Exi = Exisulind. (**D**) *In vivo* sections at 2 weeks after injury demonstrated that the transfer of retroviral vector expressing active form of PKG (PKG IαS65D and PKG IßS80D) showed the similar effect of Exisulind-treated group, which was reversed by the transfer of retroviral vector expressing dominant-negative form of PKG (PKG IαK390R and IßK405R). Scale bar = 100 or 50 μm. In case of intimal area, *P = 0.02 vs. Mock (retroviral vector only expressing CMV promoter and green fluorescent protein, GFP) in control group, **P < 0.01 vs. Mock in Exisulind-treated group. In case of I/M ratio, *P = 0.04 vs. Mock (retroviral vector only expressing CMV promoter and green fluorescent protein, GFP) in control group, **P < 0.01 vs. Mock in Exisulind-treated group, n = 10 in each group.

To test whether Exisulind shows its effect through PKG pathway, we conducted several additional experiments with PKG inhibitor (8-Rp-cPT-cGMP), or gene transfer of dominant-negative (inactive form) PKG. As shown in Fig. 4A,B, PKG inhibitor reversed Exisulind-induced PKG activation in terms of VSMCs viability and apoptosis. Western blot for phospho-VASP demonstrated that Exisulind-induced PKG activation was reversed by PKG inhibitor, indicating that Exisulind regulates PKG in VSMCs (Fig. 4C). *In vivo* study showed that gene transfer of active form of PKG (PKG IαS65D and PKG IßS80D) showed the similar result to Exisulind treatment, and that gene transfer of dominant-negative PKG (PKG IαK390R and PKG IßK405R) reversed the effect of Exisulind, suggesting that Exisulind reduced neointimal formation via PKG pathway (Fig. 4D).

### Exisulind facilitated re-endothelialization after vascular injury via PKG pathway

**Figure 5:**
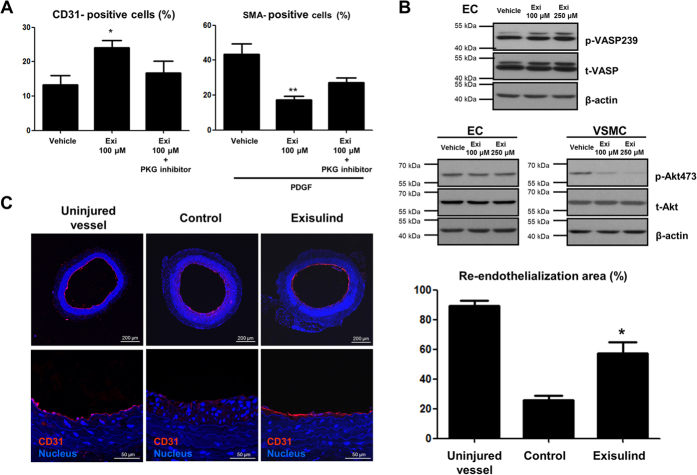
Facilitated EC-lineage differentiation and re-endothelialization by Exisulind treatment. (**A**) After three weeks of culture with peripheral blood mononuclear cells, Exisulind treatment induced the increased proportion of CD31-positive cells (EC differentiation marker), which was reversed by PKG inhibitor (8-Rp-cPT-cGMP: 20 μM). In contrast, Exisulind treatment reduced the number of cell positive for α-SMA (VMSC differentiation marker). Exi = Exisulind. *P = 0.03 vs. Vehicle in CD31-positive cells, **P < 0.01 vs. Vehicle in SMA-positive cells. (**B**) Western blot analysis for phospho-VASP and phospho-Akt in ECs and VSMCs suggests that Exisulind treatment did not reduce Akt activity in ECs but not in VSMCs. p-VASP239 = phospho-VASP at serine239, t-VASP = total VASP, p-Akt 473 = phospho-Akt at serine473, t-Akt = total Akt. (**C**) Immunofluorescence staining for CD31 in the sections at 1 week after injury. Compared to control group, Exisulind treatment increased CD31-positive cell lining. Scale bar = 100 or 50 μm. *P < 0.01 vs. Control. The data are presented as mean + SEM of five independent experiments.

Re-endothelialization can be divided into two processes such as the differentiation of vascular progenitor cells (VPCs) or endothelial progenitor cells (EPCs) into endothelial cells and the proliferation of the pre-existing endothelial cells. In terms of VPC differentiation, Exisulind treatment enhanced endothelial-lineage differentiation of human peripheral blood mononuclear cells, whereas reduced VSMC-lineage differentiation, which was reversed by PKG inhibitor (Fig. 5A). Interestingly, although Exisulind increased PKG activity in ECs just like that in VSMCs, it had different effect of Akt activity in ECs and VSMCs (Fig. 5B). This supports the results shown in Fig. 2B that endothelial cells were not affected by Exisulind treatment in contrast to the VSMCs that showed reduction of proliferation and induction of apoptosis by Exisulind. We compared the degree of re-endothelialization among groups such as uninjured, injured control, and Exisulind treatment group at 7 days after injury (Fig. 5C). Compared to the control group, Exisulind-treated group showed much higher degree of endothelialization, showing the similar.

### Exisulind treatment inhibited platelet aggregation via PKG pathway

**Figure 6:**
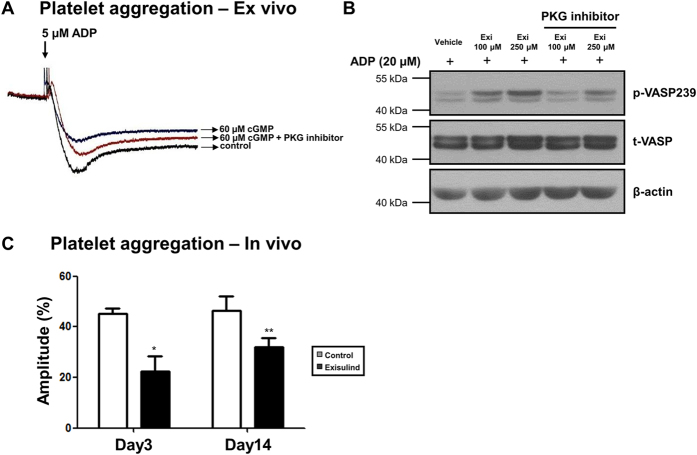
Inhibition of platelet aggregation by Exisulind. (**A**) Platelet aggregometer with platelet rich-plasma from a healthy donor showed that ADP-induced platelet aggregation was reduced by PKG activation using cGMP. Reduced platelet aggregation by PKG activation was reversed by PKG inhibitor (8-Rp-cPT-cGMP: 20 μM). (**B**) Western blot for VASP phosphorylation (indicates inhibitory activity of platelet aggregation) demonstrated that Exisulind treatment increased VASP phosphorylation and that PKG inhibitor (8-Rp-cPT-cGMP: 20 μM) reversed this effect. p-VASP239 = phospho-VASP at serine239, t-VASP = total VASP. Exi = Exisulind. (**C**) Platelet aggregometer with platelet rich-plasma from rat peripheral blood treated with vehicle or Exisulind for 3 days or 2 weeks. *P = 0.02 vs. Control at Day 3, **P = 0.05 vs. Control at Day 14. The data are presented as mean + SEM of five independent experiments.

To test whether PKG pathway is associated with platelet aggregation, platelets were treated with cGMP or cGMP plus PKG inhibitor. As shown in Fig. 6A, PKG activation with cGMP treatment reduced platelet aggregation, which was reversed by PKG inhibitor. Also in platelets (Fig. 6B), Exisulind increased the activity of PKG. *In vivo* study demonstrated that ADP-induced platelet aggregation was reduced in Exisulind-treated group compared to vehicle-treated group (Fig. 6C). These results suggest that Exisulind can inhibit platelet aggregation via PKG pathway.

## Discussion

In DES era, the drugs used in DES have been successful in reducing restenosis rate after PCI^1^,^2^. However, the issues for stent thrombosis and delayed re-endothelialization have emerged^2^,^3^. Therefore, three important points of the ideal drugs for DES are the ability of reducing restenosis, no increase of stent thrombosis, and facilitating re-endothelialization. In this aspect, Exisulind could be one of the candidate drugs satisfying those requirements.

In the present study, *in vitro* studies showed that activation of PKG by Exisulind modulated VSMC phenotype, resulting in the reduction of VSMC viability and migration. In addition, *in vivo* experiments demonstrated that Exisulind regulated VSMC phenotype and reduced neointimal formation after balloon injury via PKG activation. Interestingly, Exisulind did not change EC viability in contrast to its effect on VSMCs. Moreover, Exisulind enhanced the differentiation of EPCs into ECs through PKG activation. Finally, PKG activation by Exisulind treatment also inhibited platelet aggregation.

### Diverse mechanisms of PKG pathway in inhibiting restenosis

PKG controls the growth and differentiation of several cell types, including neuronal cells^25^, osteoblasts^26^, and VSMCs^27^. PKG plays an important role in modulating the VSMC phenotype in response to injury^13^,^14^,^15^,^28^. In our *in vitro* and *in vivo* studies, Exisulind inhibited the transition of VSMC from a contractile form to a synthetic form. Moreover, PKG inhibitors or dominant-negative PKG reversed the effect of Exisulind, suggesting that Exisulind regulates VSMC phenotype through PKG pathway. Because a synthetic form of VSMC contributes to neointimal formation, Exisulind could reduce neointimal hyperplasia after balloon injury by reducing synthetic VSMC phenotype via PKG pathway. In addition, it is known that VASP phosphorylation at serine239 inhibits VSMC proliferation^29^. Because Exisulind increased VASP phosphorylation at serine239, it showed the potent inhibitory effect on the proliferation of VSMCs, resulting in reducing neointimal hyperplasia. In our study, migration assay demonstrated that Exisulind inhibited the migration capacity of VSMCs, which is also involved in neointimal formation. Taken together, these diverse mechanisms of PKG activation might contribute to the potent effect of Exisulind on reducing restenosis.

### Exisulind shows differential effects on cell viability in VSMCs and ECs

**Figure 7:**
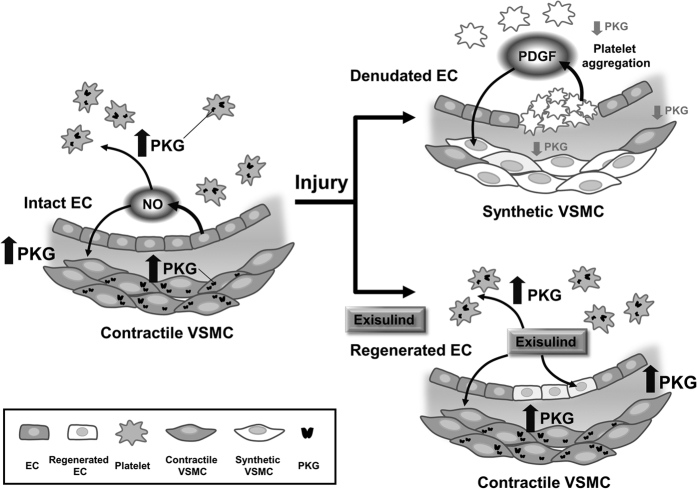
Schematic figure of the role of PKG in the vascular injury. In the uninjured vessels, intact endothelial cells produce NO, which activates PKG in VSMCs and platelets, leading to inhibition of proliferation and aggregation. After vascular injury, EC denudation decreases NO production, resulting in the decreased level of PKG in VSMCs and platelets. In addition, activated platelets produce PDGF which can activate VSMCs. All these processes contribute to VSMC proliferation and platelet activation. In contrast, Exisulind treatment can prevent the decrease of PKG activity in VSMCs and platelets, leading to inhibition of both cells in conjunction with facilitating re-endothelialization.

In order to reduce restenosis after implantation of DES, drugs eluted from stents should inhibit VSMC proliferation and facilitate endothelial regeneration. In this regard, drugs for DES need to diminish any inhibitory effects on EC viability. In this study, Exisulind reduced the viability and increased the apoptotic fraction of VSMCs, but not of ECs. One reason for this might be different responsiveness to the NO/cGMP/PKG pathway between VSMCs and ECs (Fig. 7). It is known that the NO/cGMP pathway inhibits VSMC proliferation^30^,^31^, but has an opposite effect in ECs^32^. As mentioned before, because VASP phosphorylation at serine239 by PKG inhibits VSMC proliferation^29^, Exisulind increased VASP phosphorylation via PKG activation, resulting in the reduction of VSMC viability. In contrast, in terms of ECs, PKG activation has been known to increase EC proliferation or to promote angiogenesis^33^,^34^,^35^. Therefore, by activation of the NO/cGMP/PKG pathway, Exisulind could exert an inhibitory effect on VSMC viability, but not on EC viability. The other possibility is the nuclear translocalization of PKG, although this is not a well-known mechanism. Some studies showed that PKG can phosphorylate other proteins or transcriptional factors after nuclear translocation through the exposed nuclear localization signal^36^. VSMC and EC may have different nuclear localization responses to Exisulind treatment. However, to verify these hypotheses, further experiments are needed.

### Exisulind facilitates the differentiation of Vascular Progenitor Cells (VPCs) into EC-lineage cells

It is known that NO/cGMP/PKG pathway is involved in angioblast formation and differentiation into arterial endothelium^35^. *In vivo* studies showed that re-endothelialization was increased in the Exisulind-treated group. This result might be due to an increased survival of ECs by Exisulind. In peripheral blood, there are VPCs that can be differentiated into either ECs or VSMCs^37^,^38^. *In vitro* study by FACS analysis demonstrated that Exisulind treatment induced the differentiation of VPCs into ECs. In contrast, Exisulind inhibited the differentiation of these cells into VSMCs.

### Exisulind prevents platelet aggregation

In a clinical setting, stent thrombosis has become a hot issue in the DES era. Compared to bare-metal stents, the use of DES is known to increase the incidence of stent thrombosis^3^. Regarding this point, inhibition of platelet aggregation is very important. According to our results, Exisulind decreased the aggregation of platelets. Particularly, PKG activation by Exisulind increased the VASP phosphorylation which is known to be the best marker for inhibition of platelet aggregation^39^. Therefore, in addition to reducing restenosis, Exisulind could also decrease platelet aggregation. These results imply that Exisulind has additional beneficial effects as a candidate drug for DES.

### Role of PKG in the VSMCs, ECs, and Platelets after Vascular Injury

In the uninjured vessels, intact endothelial cells produce NO, which activates PKG in VSMCs and platelets. Activated PKG prevents VSMC proliferation and platelet aggregation (Fig. 7). After vascular injury, the denudated EC decreases NO production, resulting in the decreased level of PKG in VSMCs and platelets. In addition, activated platelets produce PDGF which can activate VSMC proliferation. All these processes contribute to VSMC proliferation and platelet activation. In contrast, Exisulind treatment can prevent the decrease of PKG activity in VSMCs and platelets after vascular injury. This activation of PKG inhibits VSMC proliferation and platelet aggregation, in conjunction with facilitating re-endothelialization.

When we consider the pharmacokinetics of the eluted drug in vasculature after implantation of DES, the concentration of the drug increases at early phase whereas it decreases thereafter after reaching peak. Considering this point, Exisulind could be more ideal for DES. According to the results of our study, Exisulind inhibited the proliferation of VSMCs in a dose-dependent manner. However, in terms of VPC differentiation, the beneficial effect of Exisulind can be seen even at a low concentration (100 μM). We can assume that in the initial phase of stent implantation, the main effect of high concentration Exisulind might be inhibition of VSMC proliferation, and that in the later phase, at a low concentration, it might mainly induce differentiation to EC-lineage. Therefore, it would be ideal if Exisulind could exert different effects sequentially at each phase after implantation of Exisulind-eluting stents.

In term of the effect of Exisulind on the induction of apoptosis, we can assume that the activation of PKG might induce the activation of JNK pathway via phosphorylation of MEKK1 as seen in cancer cells. It is known that JNK activation play an important role in the process of apoptosis such as cleavage of caspase^40^. As a high concentration of Exisulind is required to induce apoptosis in cancer cells, we can see that, in inducing apoptosis in VSMCs, a high concentration of Exisulind such as 250 μM is also needed. In human, with the 400 mg bid dosage, the blood level of Exisulind is known to be from 18 to 48 μM^41^, which is lower than the level of Exisulind used in the usual *in vitro* experiments. As discussed above, however, if we consider the pharmacokinetics of the eluted drug after the stent implantation, the level of Exisulind around vessels might be up to that of *in vitro* studies.

In conclusion, our data showed that Exisulind is a potent inhibitor of neointimal formation by PKG activation. Moreover, Exisulind prevents platelet aggregation and increase re-endothelialization via PKG pathway. These findings suggest that Exisulind could be a new candidate drug for DES for the prevention of restenosis without unfavorable events.

## Additional Information

**How to cite this article**: Kim, J.-Y. *et al*. Activation of Protein Kinase G (PKG) Reduces Neointimal Hyperplasia, Inhibits Platelet Aggregation, and Facilitates Re-endothelialization. *Sci. Rep.* **6**, 36979; doi: 10.1038/srep36979 (2016).

**Publisher’s note:** Springer Nature remains neutral with regard to jurisdictional claims in published maps and institutional affiliations.

## Supplementary Material

Supplementary Information
