# Supplementary material for: Activation of Protein Kinase G (PKG) Reduces Neointimal Hyperplasia, Inhibits Platelet Aggregation, and Facilitates Re-endothelialization
Source: Sci Rep. 2016 Nov 11;6:36979. doi: 10.1038/srep36979 (PMC5105062; doi:10.1038/srep36979)
Supplement: Supplementary Information [file srep36979-s1.doc]

**Online Data Supplements for**

**Activation of PKG Reduces Neointimal Hyperplasia, Inhibits Platelet Aggregation, and Facilitates Reendothelialization**

Ju-Young Kim^1,3,5^, Han-Mo Yang^1,2,3,5^ Joo-Eun Lee^1,3,5^, Baek-Kyung Kim^1,3^, Sooryeonhwa Jin^1,3^, Jaewon Lee^1,3^, Kyung-Woo Park^1,2,3^, Hyun-Jai Cho^1,2,3^, Yoo-Wook Kwon^1,3^, Hae-Young Lee^1,2,3^, Hyun-Jae Kang^1,2,3^, Byung-Hee Oh^1,2,3^, Young-Bae Park^1,2,3^, Hyo-Soo Kim^1,2,3,4^

^1^National Research Laboratory for Cardiovascular Stem Cell, Seoul National University College of Medicine, Seoul, Republic of Korea

^2^Department of Internal Medicine, Seoul National University Hospital, Seoul, Republic of Korea

^3^Innovative Research Institute for Cell Therapy, Seoul National University Hospital, Seoul, Republic of Korea

^4^Molecular Medicine and Biopharmaceutical Sciences, Seoul National University, Seoul, Korea

^5^These authors contributed equally to this work.

Correspondence : Hyo-Soo Kim, MD/PhD. FAHA

 Director of Cardiac Catheterization Laboratory & Coronary Intervention

Director of National Research Laboratory for Cardiovascular Stem Cell

 Professor, Department of Internal Medicine,

Seoul National University Hospital,

101 Daehak-ro, Jongro-gu, Seoul 110-744, Korea

Tel : 82-2-2072-2226

Fax : 82-2-766-8904

E mail : hyosoo@snu.ac.kr , usahyosoo@gmail.com

This supporting material contains:

**Supplemental figure 1 with figure legends**

**Supplemental Figure S1**

**Supplemental Figure Legends**

**Figure S1. Characterization of endothelial cells (ECs) and vascular smooth muscle cells (VSMCs) cultured from rat aorta.**

A) Confluent monolayers of EC showed typical “cobblestone” morphology. Then, cultured cells were fixed with cold methanol and stained with VE-cadherin. VE-cadherin staining was observed in adherent junctions of ECs (Green = VE-cadherin, Blue = DAPI). B) Confluent rat VSMCs were stained with calponin. Scaled down image in the right panel is α-SMA immunostaining (Red = Calponin, Blue = DAPI, Green = α-SMA). Scale bar = 20µm.
